# iSDAsoil: The first continent-scale soil property map at 30 m resolution provides a soil information revolution for Africa

**DOI:** 10.1371/journal.pbio.3001441

**Published:** 2021-11-01

**Authors:** Matthew A. E. Miller, Keith D. Shepherd, Bruce Kisitu, Jamie Collinson

**Affiliations:** 1 Innovative Solutions for Decision Agriculture Ltd (iSDA), Harpenden, United Kingdom; 2 World Agroforestry Centre (CIFOR-ICRAF), Nairobi, Kenya

## Abstract

Recent developments in remote sensing and machine learning have allowed African soil properties to be mapped at an unprecedented resolution of 30 m. iSDAsoil is an open access resource that will empower the community to embrace ’hyperlocal’ approaches such as field-level agronomy advice for smallholder farmers.

## Teaser

Open access, high-resolution soil property maps have been created for Africa at 30 m resolution, using machine learning trained on over 100,000 analysed soil samples. Combined with other field-level information, iSDAsoil enables the possibility of site-specific agronomy advisory for smallholder farmers.

## Why should we care about soil?

Soil is an often overlooked resource, but its importance in our everyday lives should not be underestimated. Plants are the basis of life on earth but depend on a thin layer of soil to grow. Soils are of particular importance to our food production systems. A healthy soil not only supplies nutrients, but also water to support plant growth. Soils are essential components of the global cycle of nitrogen, carbon, and phosphorus, as well as filtering waste and pollution. Healthier soils can also capture more carbon and help in the fight against climate change. In short, soil health and human health are inextricably linked.

## Soil information in Africa

Africa is home to a strikingly diverse set of soils [1]; however, high-resolution, continent-wide soil information in actionable form has historically been lacking. Mismanagement of soils due to a lack of soil information can degrade the soil and limit crop yields. Farmers that invest in a fertiliser that is poorly tailored to soil conditions can find themselves out of pocket and cause damage to the environment. To address this gulf in information, the African Soil Information Service (AfSIS) project was created. The AfSIS project developed a consistent methodology for sample collection and analysis of soil chemical and physical properties. Using this methodology, AfSIS collected and analysed a consistent dataset of around 40,000 soil samples across more than 15 countries, representing diverse environments across sub-Saharan Africa.

## From soil data to soil maps

While the AfSIS approach revolutionised many aspects of soil data collection at scale, advances have also been made in the area of Digital Soil Mapping (DSM; [2]). DSM aims to understand the relationship between lab-measured or field-observed soil properties and the surrounding measurable environment, incorporating climatic, geological, and remote sensing data. Predictive models are then trained to generate soil maps at a consistent geospatial basis. Advances in machine learning algorithms and the widespread availability of high-resolution satellite data have driven increases in soil map resolution, as well as improved accuracy at predicting many soil properties that are important for crop growth [3].

## Introducing iSDAsoil

While the previous state-of-the-art maps for Africa are at 250 m resolution [3], this spatial resolution is not adequate for site-specific, field-level advice. Many farms are smallholdings, and fields are smaller and more heterogenous when compared with farms in other continents such as North America. iSDAsoil is the world’s first continental-scale soil property map at 30 m resolution and provides soil information at a resolution that approaches the true variability on the ground [4]. For technical information on the workflow used, please visit our Technical Information page. iSDAsoil provides estimations for over 20 different soil properties, including an implementation of the Fertility Classification Framework [5]. A selection of over 100,000 geolocated soil samples were used for model training, collected across more than 10 different datasets. An ensemble machine learning approach was used, alongside an extensive collection of more than 100 covariate layers [4] to predict soil properties for more than 24 billion pixels.

This soil information, coupled with additional site-specific information, can provide the basis for field-level tailored agronomy advisory for smallholders (see Box 1). iSDAsoil is a completely open access soil resource, and regular updates are planned, such that the maps can be improved when additional soil or satellite data becomes available, or improved methodologies are developed. The iSDAsoil maps can be browsed at https://isda-africa.com/isdasoil.

## Advances in spatial and spectral resolution

The increase in map resolution has been driven by the incorporation of high-resolution satellite data from Sentinel 2 and Landsat Satellites, both of which provide images at 30 m or finer resolution. A number of different spectral wavelength bands from the Sentinel 2 satellite were important for predicting soil properties, including shortwave infrared (bands 9, 11, and 12; [4]). While these wavelengths are well known for monitoring of vegetation, their importance in predicting soil properties was previously less clear. For those not closely involved in soil mapping, it might come as a surprise that we are able to learn about the soil chemical and physical properties by measuring reflected light from outer space. This is due to soils directly reflecting light at different wavebands as well as environmental correlation between soil properties and vegetation/terrain properties that can be observed from space.

## The importance of uncertainty

**Fig 1:**
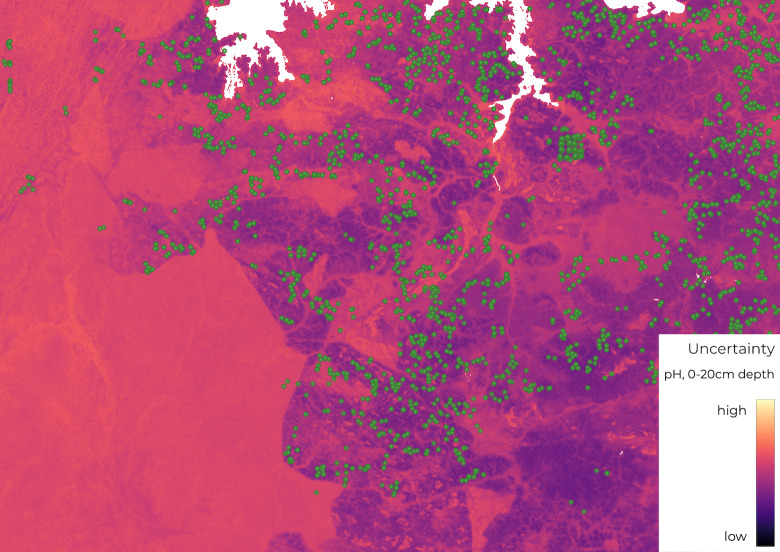
The effect of soil sampling density on the uncertainty of the resultant soil maps of pH, for an area in Northern Tanzania. Green symbols highlight locations of training points used for iSDAsoil model training. An area of higher uncertainty (bottom left) highlights the importance of a consistent set of training points.

Another important breakthrough of iSDAsoil is the per-pixel estimation of uncertainty that is provided alongside the data. For each soil property, 2 maps are created—a “data” map and an “uncertainty” map. This allows users to understand for any given location, with what level of confidence the soil property prediction has been made. This can inform about locations where there is a high degree of uncertainty in the soil properties, which might help to target new soil sampling campaigns (see Fig 1). The new data could then be incorporated to produce more accurate predictions for these locations. A knowledge of the uncertainty also guides the confidence level with which soil-based recommendations can be made.

Nevertheless, it is important to be aware of the bias in soil sampling locations. The majority of sampling points have been collected from agricultural land and validated against similarly located points. Therefore, predictions (and uncertainty calculations) for locations that are not well represented in the training set (such as dense forest) are likely to be less accurate. Currently, the maps are also “static” and therefore represent soil properties around the time of the sampling dates (the majority of which were collected between 2000 and 2020), which may have subsequently changed. This issue can be somewhat addressed by collecting additional field-level information (see Box 1) and continual updating with new samples.

## Applications of iSDAsoil

Many farmers might not be able to make use of the soil data directly but can benefit from tailored advice that uses soil maps, such, for example, a fertiliser decision support tool. Modelling approaches, such as Bayesian networks and Monte Carlo simulations, can incorporate data with uncertainty to produce a possible range of outcomes, producing a far more reliable result than feeding models with input data based upon solely averages [6]. Incorporating uncertainty also allows the risk associated with recommendations to be presented to farmers. iSDA has pioneered this approach in a nutrient management advisory tool, the Virtual Agronomist, which is robust under missing or uncertain data (see Box 1).

In addition to site-specific fertiliser interventions, iSDAsoil can contribute to more regional-scale activities, including liming campaigns (to address acidic soils), regional fertiliser blend formulation and demand forecasting, and soil conservation planning. An understanding of the soil can also help to alleviate nutrient deficiencies in humans (geonutrition), by ensuring the crop type most suited to the soil is grown.

## How to improve iSDAsoil

iSDAsoil is planned to be a dynamic resource: It is hoped that in making the maps open access, the community will be encouraged to contribute additional soil data, allowing for regular updates to create increasingly accurate maps. iSDA is also planning to incorporate modelling improvements to reduce bias and uncertainty. If you would like to contribute soil data, or learn more about the Virtual Agronomist, please get in touch at info@isda-africa.com.

## Accessing the data

The iSDAsoil data are completely open access under a CC-BY 4.0 licence and can be browsed at the iSDAsoil homepage. To access the data, users can sign up to the free API that allows retrieval of soil properties by latitude/longitude. Entire files (or subsets thereof) can be downloaded via the AWS Registry of open data listing. The data are also available via Google Earth Engine.

**Box 1. The view from the ground**

iSDA is developing a smartphone app, the Virtual Agronomist, for smallholder coffee farmers in Kenya to understand the status of their soil health and get a tailored fertiliser recommendation. The Virtual Agronomist combines iSDAsoil with context-specific information provided by the farmers and is delivered through intermediaries called Village Enterprise Advisors (VEAs). These are young and energetic rural entrepreneurs trained to use the Virtual Agronomist. The limited availability of accessible farmer decision support tools and agronomists has created an opportunity for the VEAs to deliver the service as a business. The aim is to optimise fertiliser application and ultimately increase the profitability of farmer enterprises.

## Abbreviations

AfSIS: African Soil Information Service
DSM: Digital Soil Mapping
VEA: Village Enterprise Advisor
